# Unique Acute Phytophotodermatitis Reaction and a Visual Diagnostic Guide

**DOI:** 10.7759/cureus.59199

**Published:** 2024-04-28

**Authors:** Iya A Agha, Sarah Montaquila, Hasan Alobaidi

**Affiliations:** 1 Dermatology, New York Institute of Technology College of Osteopathic Medicine, New York, USA; 2 Interventional Radiology, New York Institute of Technology College of Osteopathic Medicine, New York, USA; 3 Internal Medicine Department, Jamaica Hospital Medical Center, New York, USA

**Keywords:** solar radiation, photoreaction, contact dermatitis, dermatology, dermatitis, phytophotodermatitis

## Abstract

Here, we describe a case of a 48-year-old caucasian female with no significant past medical history who presented to the outpatient dermatology clinic with an extremely painful and significant phytophotodermatitis (PPD) reaction to over 30% of her body surface area. The patient presented after partaking in a ritualistic ceremony where she was exposed to plant and citrus juices and subsequently sunbathed while on a tropical vacation. While not an infrequent diagnosis, this patient presented to the dermatologist in extreme pain after having no education on avoiding such triggers. This diagnosis is relatively underdiagnosed and leads to a lack of patient counseling on the hazards of UV exposure secondary to contact with certain plant and fruit juices. Lack of patient awareness leads to an increase in disease burden. Furthermore, this patient suffered a large body surface area reaction in contrast to the pathognomonic description of phytophotodermatitis secondary to the exposure to lime juice which causes relatively less total body surface area exposure.

## Introduction

Phytophotodermatitis is a phototoxic reaction that occurs after exposure to certain plant chemicals known as furanocoumarins to the skin. Specifically, long-wave ultraviolet radiation from the sun activates the furocoumarins, a substance found in some plants such as citrus, fig trees, parsnip, fennel, celery, parsley, and giant hogweed, which results in a photochemical reaction causing cell membrane damage to skin cells [1-3]. The reaction presents within 24 hours with painful damage and inflammation to the sun-exposed skin [1,4]. The severity of the damage can vary depending on the amount of exposure to the botanical chemical agent and exposure time to ultraviolet radiation, and the presentation can vary from an erythematous rash similar to sunburn to bullae formation with eventual post-inflammatory skin hyperpigmentation in sun-exposed areas [1,2]. Exposure to plant chemicals in reported cases has been from the smearing of plant sap or exposure of citrus juice onto the skin, followed by light exposure [2,3]. Most cases of phytophotodermatitis occur in the summer months when there are higher levels of ultraviolet radiation or during tropical vacations with high sun exposure [1].

The diagnosis of phytophotodermatitis is clinical and based on a thorough history of exposure to plant chemicals and sunlight. Differential diagnoses include contact or allergic dermatitis, burns, or cellulitis. Management of phytophotodermatitis consists mainly of supportive measures, including cool compresses and pain management, topical antiseptics to prevent infection, topical steroid creams, and patient education and trigger avoidance. In severe cases, a dermatologist referral may be necessary.

## Case presentation

**Figure 1:**
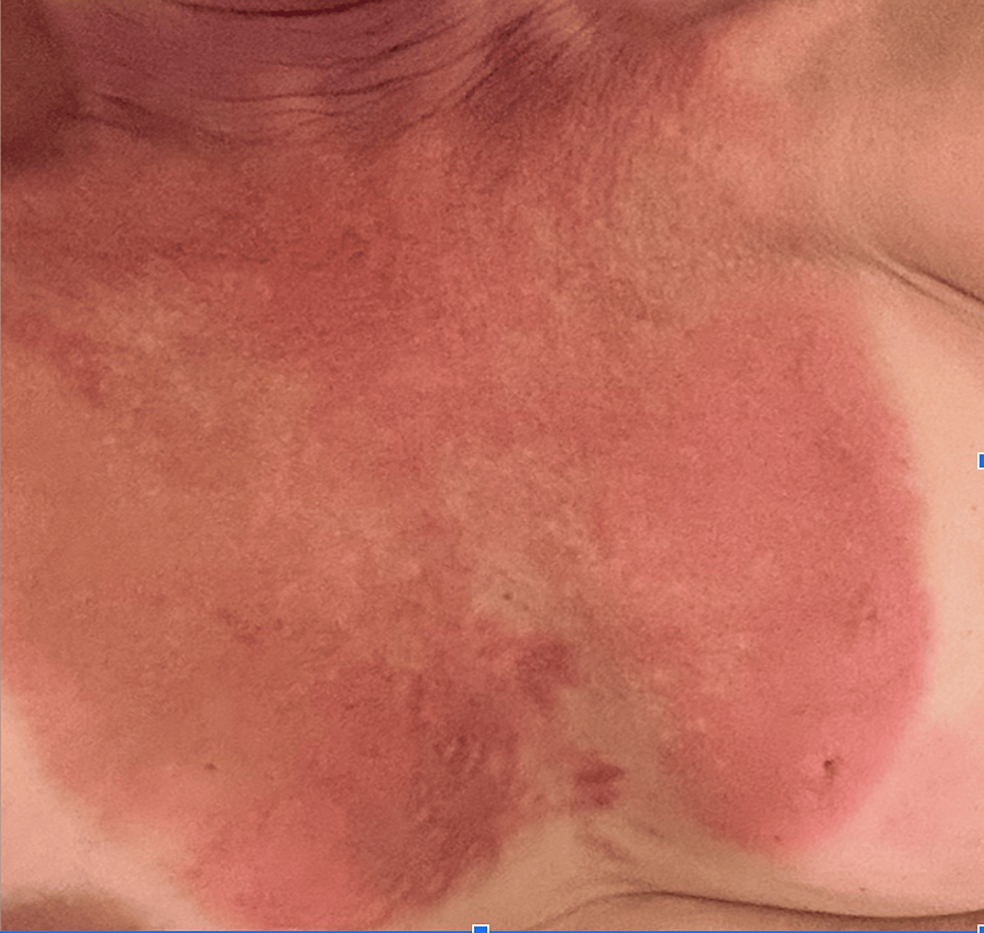
Image of the patient's chest at presentation Anterior chest erythema demonstrating extensive surface area inclusion and demarcation of bathing suit line representing solar reaction.

**Figure 2:**
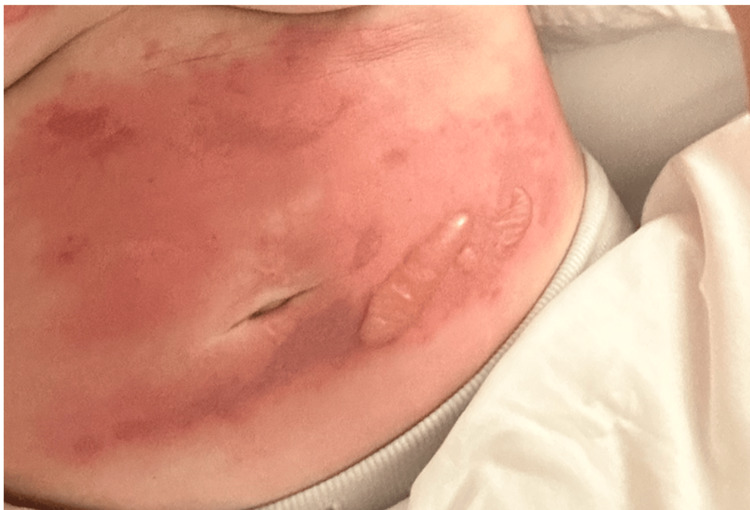
Image of the patient's abdomen at presentation Extensive erythema and blistering with a positive Nikolsky sign on the anterior abdomen.

A 48-year-old caucasian female with no past medical history presented to the out-patient dermatology clinic with new onset exquisitely painful, blistering, erythematous skin on her abdomen, chest, neck, and chin that gradually appeared and increased in severity over the course of a three-day period. Physical examination revealed closed vesicles located on her left lower quadrant, overlaying erythematous, tender skin, and a hyperpigmented reaction on her entire anterior chest and abdomen (Figures 1, 2). 

Further history revealed that the patient recently returned from a vacation in Mexico where she partook in a “sweat lodge,” where she was subjected to plant substance being placed on her body. The patient stated that after she finished in the sweat lodge, she swam in the ocean and proceeded to lay in the sun for approximately 30 minutes. The next morning, the patient reported a sunburn-like reaction present on her abdomen and chest but no further complaints. The patient returned home and, over three days, noticed an increase in the reaction and the formation of the vesicles described previously. The patient’s pain increased to its highest intensity being a 9 out of 10 on the pain scale. 

The patient was seen in the outpatient dermatology clinic, where she was diagnosed with phytophotodermatitis secondary to plant exposure. She was given topical triamcinolone acetonide 0.1% cream to apply twice a day (BID) and oral prednisone 10 mg taper course with subsequent improvement of pain and symptomatology. The patient’s reaction initially resembled a contact dermatitis presentation, and her diagnosis depended on proper history-taking and attention to visual diagnostic skills.

## Discussion

The pathophysiology of phytophotodermatitis (PPD) begins when photosensitizers are applied to the skin before the dermis is exposed to UV, leading to photo-contact dermatitis that may mimic other common dermatologic pathologies such as allergic contact dermatitis, cellulitis, or certain fungal infections [4]. The reaction in itself is believed to be due to the photosensitizing furocoumarins that are present in many plants in their branches, leaves, and residue, which is probably due to the proteolytic plant defense mechanism that depends on the enzymatic activation as self-protection in the plant (Table 1). When in contact with the skin, the unique enzymatic activation leads to irritation of the skin and eventual phototoxic reactions. Upon subsequent exposure to ultraviolet light A (UVA) at 320~400 nm exposure, there is extensive reactive oxygen species generation and dermal damage [6].

**Table 1:** Common plants that cause phytophotodermatitis A non-exhaustive list of common plant irritants that can cause phytophotodermatitis [5].

Plant Type (Family Name)	Common Name(s)
Moraceae*	Fig*
Umbelliferae	Parsley, Carrot, Fennel, Celery, Giant Hogweed
Rutaceae*	*Lime, *Lemon, Bitter orange, Burning Bush
Hypericaceae	St. John’s Wort

While PPD can be diagnosed on a clinical basis, Hematoxylin and Eosin (H&E), staining of the phototoxic reactions illustrate sub-epithelial blister and intensive epidermal necrosis [4]. This reaction improves clinically with diagnosis and treatment with systemic and topical corticosteroids; however, secondary sequelae can lead to morbidity in patients who may suffer from post-inflammatory hypopigmentation that may be more permanent. Furthermore, in any patient with a damaged skin barrier, there is an increased risk for secondary infections that may be more likely if there is exposure to irritants and bacteria that may be more likely due to the initial presentation due to secondary exposure to external irritants. Therefore, patients should be counseled on monitoring for symptoms that could point to secondary infection, including increased erythema, pain, and fever, when resolution of symptomatology should already be underway.

The mechanism of pathology raises alarm bells as patients may be increasingly exposed to activities that facilitate a phototoxic reaction, including but not limited to similar ritualistic behavior through sweat lodges, home/folk remedies utilizing plant properties that may be understudied, and the well-known lime juice from someone making a drink before sun exposure among others. With the increase in health-focused trends that may promote the aforementioned risk factors [7]. Proper patient education in a clinical setting may help reduce incidence and misinformation that could propagate in patient populations that are susceptible to trends in dermatology.

While similar to the presentation of allergic contact dermatitis, a delayed hypersensitivity reaction, PPD can be differentiated through presentation in that it only presents in UV-exposed areas [8]. In addition, PPD heals often with signs of hyperpigmentation, whereas contact dermatitis usually heals without hyperpigmentation or hypopigmentation [9].

It is exceedingly important that dermatologists are aware of the potential diagnosis to avoid unnecessary patient exposure to treatments for incorrect diagnoses, such as unwarranted antibiotic use in assumed cellulitis instead of the recommended corticosteroids that improve this pathology. Through proper history taking, attention to possible travel history, and maintaining an attuned critical thinking, correct diagnosis and treatment can be easily attained [3]. 

## Conclusions

In conclusion, here we present a case of significant phytophotodermatitis in a 48-year-old caucasian female with no significant past medical history presenting after solar exposure and chemical exposure in a sweat lodge. Proper patient education and timely diagnosis and treatment influence patient outcomes and secondary sequelae in phytophotodermatitis cases. PPD can present on a scale of minimal exposure to solar radiation secondary to incrementally smaller amounts of plant and fruit products to extreme chemical burns secondary to a larger body surface area exposure and reaction, as seen in this patient. Secondary sequelae of PPD range from almost complete clinical resolution to secondary infections and permanent cutaneous damage causing hyper or hypopigmented scarring. While not uncommon in presentation, a lack of timely diagnosis due to underdiagnosis can prevent treatments that may reduce the secondary sequelae. Physicians and patients will benefit from proper patient education on the avoidance of triggers coupled with a widespread greater understanding of the diagnosis. 
